# Natural genetic variation in *C. elegans* identified genomic loci controlling metabolite levels

**DOI:** 10.1101/gr.232322.117

**Published:** 2018-09

**Authors:** Arwen W. Gao, Mark G. Sterken, Jelmi uit de Bos, Jelle van Creij, Rashmi Kamble, Basten L. Snoek, Jan E. Kammenga, Riekelt H. Houtkooper

**Affiliations:** 1 Laboratory Genetic Metabolic Diseases, Amsterdam UMC, University of Amsterdam, Amsterdam Gastroenterology and Metabolism, 1105 AZ Amsterdam, The Netherlands;; 2 Laboratory of Nematology, Wageningen University and Research, 6708 PB, Wageningen, The Netherlands

## Abstract

Metabolic homeostasis is sustained by complex biological networks that respond to nutrient availability. Genetic and environmental factors may disrupt this equilibrium, leading to metabolic disorders, including obesity and type 2 diabetes. To identify the genetic factors controlling metabolism, we performed quantitative genetic analysis using a population of 199 recombinant inbred lines (RILs) in the nematode *Caenorhabditis elegans*. We focused on the genomic regions that control metabolite levels by measuring fatty acid (FA) and amino acid (AA) composition in the RILs using targeted metabolomics. The genetically diverse RILs showed a large variation in their FA and AA levels with a heritability ranging from 32% to 82%. We detected strongly co-correlated metabolite clusters and 36 significant metabolite quantitative trait loci (mQTL). We focused on mQTL displaying highly significant linkage and heritability, including an mQTL for the FA C14:1 on Chromosome I, and another mQTL for the FA C18:2 on Chromosome IV. Using introgression lines (ILs), we were able to narrow down both mQTL to a 1.4-Mbp and a 3.6-Mbp region, respectively. RNAi-based screening focusing on the Chromosome I mQTL identified several candidate genes for the C14:1 mQTL, including *lagr-1*, Y87G2A.2, *nhr-265*, *nhr-276*, and *nhr-81*. Overall, this systems approach provides us with a powerful platform to study the genetic basis of *C. elegans* metabolism. Furthermore, it allows us to investigate interventions such as nutrients and stresses that maintain or disturb the regulatory network controlling metabolic homeostasis, and identify gene-by-environment interactions.

Energy homeostasis is maintained by biological networks that are affected by nutrient availability and keep functional balance at cellular and molecular levels (Andreux et al. 2012). When this equilibrium is disrupted by genetic and/or environmental perturbations, such an imbalanced metabolic system can lead to metabolic disorders, including obesity and type 2 diabetes (Andreux et al. 2012). To study the interactions between genetic and environmental factors, different approaches are used in model organisms, including reverse and forward genetics (Williams and Auwerx 2015). Reverse genetics approaches comprise techniques that focus on the phenotypic impact of the knockdown, knockout, or overexpression of specific candidate genes. This approach typically focuses on a single gene and therefore has several limitations (Williams and Auwerx 2015): (1) The additive and nonadditive interactions between gene variants cannot be observed; (2) common variants with a subtle effect cannot be detected; and (3) prior hypotheses about the gene function are a prerequisite. Instead, forward genetics bypasses these limitations as it exploits the natural phenotypical variation in a population to identify causal genetic variants (Williams and Auwerx 2015). This involves classic mutagenesis screens and techniques such as quantitative trait loci (QTL) analysis and genome-wide association studies (GWAS). QTL analysis is a statistical technique that examines the association between a marker genotype and a quantitative trait, i.e., a trait with continuous phenotypic variation that is affected by genetic and environmental factors. For QTL analysis, a segregated population is used to find genomic regions that are associated with the trait variation in the population (Slate 2005). GWAS relies on natural populations to identify common genetic variants associated with traits and has been successful in identifying many loci associated with susceptibility to complex diseases such as type 1 and type 2 diabetes (Visscher et al. 2017). In this study, we chose to use the nematode *Caenorhabditis elegans* as a model organism to explore genetic variation affecting metabolic parameters. Considerable variation would validate *C. elegans* for further identification of complex gene by environment interactions correlated with metabolism.

*C. elegans* is a versatile model organism for understanding complex genetic pathways underlying distinct phenotypes such as stress response, lifespan, host–pathogen interaction, and behavior (Gao et al. 2017b). The Kammenga group generated a segregating population of *C. elegans* derived from the genetically and ecologically divergent strains N2 (Bristol) and CB4856 (Hawaii), which is suitable for studying genome-to-phenome relations (Li et al. 2006; Andersen et al. 2012; Sterken et al. 2015). The segregating population consists of 199 homozygous recombinant inbred lines (RILs) that have been SNP genotyped and phenotyped for several traits, including gene expression (Li et al. 2010; Rockman et al. 2010; Viñuela et al. 2010) and stress-response hormesis (favorable response to mild stress) (Gutteling et al. 2007; Doroszuk et al. 2009; Rodriguez et al. 2012; Snoek et al. 2017). The 199 RILs all have a different combination of alleles derived from the N2 or CB4856 parental strains, allowing us to determine the natural influence of genetics on a phenotypic trait.

In *C. elegans*, changes in metabolism are often studied at the transcriptional level, and multiple metabolic genes have been identified in worms (Houtkooper et al. 2013; Lopez-Otin et al. 2013; Gao et al. 2017b). At the same time, to get a comprehensive understanding of the overall metabolic changes, one has to get closer to measuring metabolic physiology, for instance using metabolomics. Based on recent advances in the metabolomics field, our group developed a sensitive mass spectrometry (MS)–based platform for measuring metabolites in *C. elegans*. This platform allows us to rapidly detect 44 medium-chain, long-chain, and very-long-chain fatty acids (FAs) (C14:0-C30:0) and 19 amino acids (AAs) in a sample of approximately 500 worms (Gao et al. 2017a). Using this platform, we measured metabolites in the RILs of *C. elegans* on a large scale and identified the effect of genetic variation on metabolism (Fig. 1). Overall, we systematically investigated the genetic basis of metabolism by QTL mapping, and based on our findings, we propose that this RIL panel is a powerful platform to study complex metabolic traits that underlie gene-by-environment interactions.

**Figure 1.**
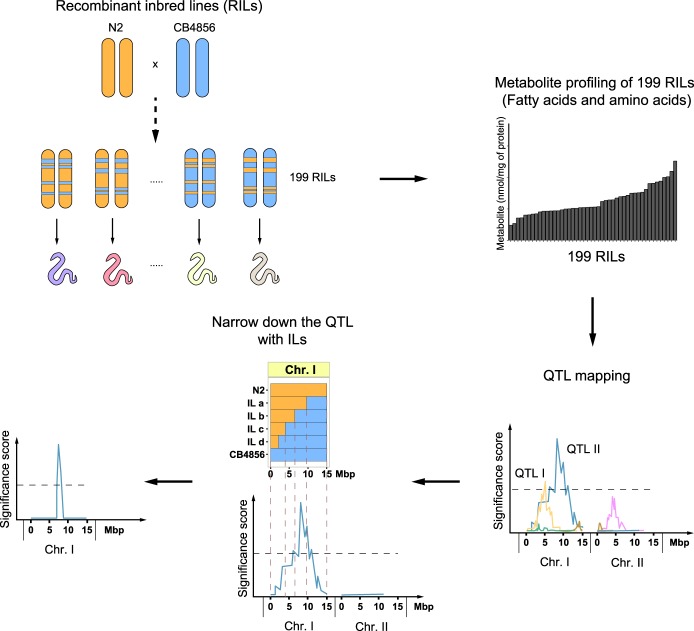
Flowchart of the strategy to identify genetic loci that control metabolic traits. Metabolite profiles for 19 amino acids (AAs) and 44 fatty acids (FAs) were measured in 199 recombinant inbred lines (RILs). These metabolite profiles were plotted and correlated with the RIL genetic map, identifying genetic loci linked to variation in metabolite levels (metabolite quantitative trait loci, mQTL). Subsequently, these mQTL were verified/confirmed and fine-mapped using introgression lines (ILs), strains containing a small locus of one parental strain in the genetic background of the other parental strain ([N2] orange; [CB4856] blue).

## Results

### Metabolite levels in *C. elegans* show high heritable variation

**Figure 2.**
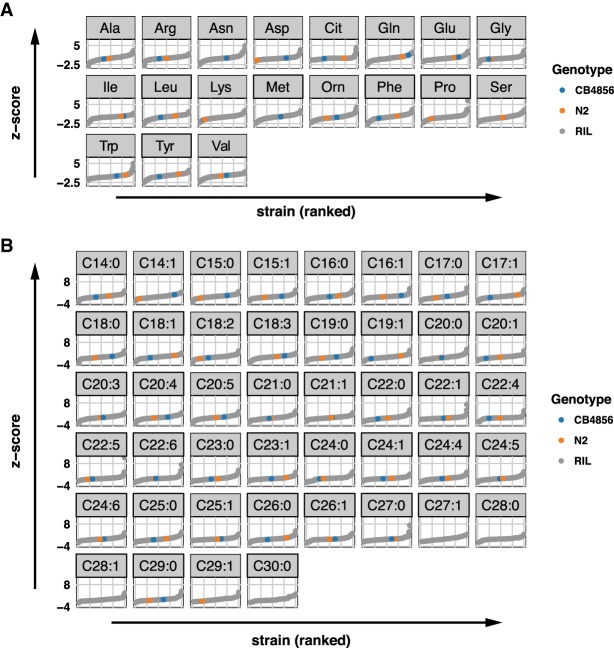
Metabolite levels across the 199 RILs and the two parental strains. (*A*) The average trait level of 19 AAs is first expressed as percentage of total AAs, followed by *z*-score transformation. The 199 RIL values are indicated in gray, the value of the parental strain N2 in orange, and the value of the parental strain CB4856 in blue. Trait levels below limit of quantification of AA measurement (0.4 nmol/mg of protein) are not shown. (*B*) The mean values of the levels of 44 FAs are shown after data transformation (the absolute FA concentration is expressed as a percentage over total FAs, followed by *z-*score transformation). The FA levels in 199 RILs are indicated in gray, those in the parental strain N2 and CB4856 are indicated in orange and blue, respectively. FA levels below limit of quantification of the measurement (0.03 nmol/mg of protein) are not shown.

To extract general principles about the complex genetics of metabolism, we measured the impact of genetic variation on metabolite levels in 199 RILs using our recently established sensitive MS platform (Fig. 1; Supplemental Table S1; Li et al. 2006; Thompson et al. 2015; Gao et al. 2017a). We chose to collect young adult worms for measuring their metabolite profiles as the metabolite amounts at this age are not influenced by egg production or aging per se (Gao et al. 2017a). The metabolite traits displayed high levels of variation across the RILs, sometimes beyond what was observed in the two parental strains, suggesting transgressive segregation (Fig. 2A,B; Supplemental Table S1). For instance, the two most abundant metabolites, C18:1 and alanine, showed a large absolute difference between the RILs with the lowest and highest abundance of 6.5- and 5.6-fold, respectively (Supplemental Table S2). In addition, metabolites present at lower concentrations, including C20:3 and methionine, showed a large absolute difference between the lowest and highest RIL of 10.5- and 11.1-fold, respectively. Transgression was observed for 18 metabolites, especially in FAs (FDR = 0.05) (Supplemental Table S3). Transgression analysis is a measure for the genetic complexity of a trait, for instance due to genetic interactions. It showcases that metabolite levels are affected by multiple polymorphic genes of which the alleles cause a balanced phenotype in the parental lines.

**Figure 3.**
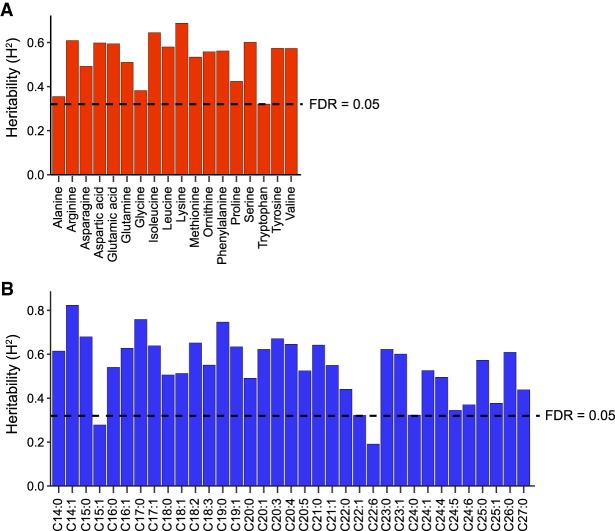
Heritability analysis of AAs and FAs. We estimated broad-sense heritability (*H*^2^) of metabolic traits based on a set of 51 RILs that were measured at least three times. The bars indicate the *H*^2^, where the dashed line indicates a permutation-based false discovery rate (FDR) of 5% (based on 1000 permutations). We found evidence for strong, significant, heritable variation in most AA (*A*) and FA (*B*) abundances, ranging from 0.32 to 0.82 (FDR = 0.05). Only for FA C15:1 and C22:6 no significant *H*^2^ was found.

Next, we calculated the broad-sense heritability (*H*^2^) of these metabolite traits, which is a measure of the genetic contribution to the variation in metabolite levels (Fig. 3). For highly heritable traits, the contributing genomic loci (quantitative trait loci, QTL) are more likely to be found. To calculate the *H*^2^, we measured metabolite levels in 51 RILs and the two parental strains an additional three times. We found significant heritability for 51 metabolite traits (FDR = 0.05), ranging from 0.32 (tryptophan) to 0.69 (lysine) for the AAs (Fig. 3A), and from 0.32 (C22:1) to 0.82 (C14:1) for the FAs (Fig. 3B; Supplemental Table S4). Together, our data show that both AAs and FAs have moderate to high heritability, increasing the chance to identify significant QTL. Specifically, the traits with nonsignificant transgression and high heritability, for instance the FA C14:1 (*H*^2^ = 0.82), are most likely associated with a single major locus explaining most of the metabolic variation.

### Metabolite levels exhibit strong correlations in the RIL cohort

**Figure 4.**
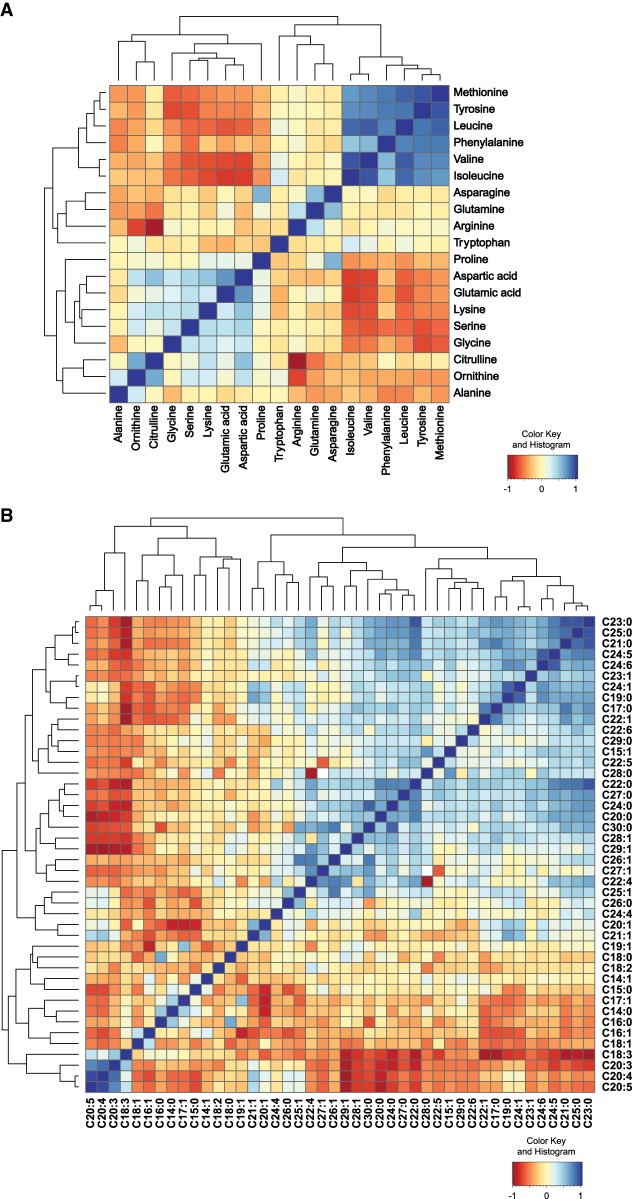
Correlation analysis of AA and FA species in the RIL strains. (*A*) Correlation heat map of AA profiles of all the RIL strains. There was a strong cluster of methionine, tyrosine, phenylalanine, arginine, and branched-chain amino acids (BCAAs) leucine, isoleucine, and valine. Another positively correlated cluster was found in ornithine, citrulline, glycine, serine, lysine, glutamic acid, and aspartic acid. The former positive cluster was negatively correlated with some AAs from the latter cluster, including proline, aspartic acid glutamic acid, lysine, serine, and glycine. (*B*) Correlation heat map of FA profiles of the RILs. Strong correlation was found in a group of long-chain and very-long-chain FAs. Polyunsaturated fatty acids (PUFAs) were also clustered and positively correlated.

Levels of different metabolites are likely correlated since homeostasis is supported by integrated metabolite networks (Houtkooper et al. 2010). We calculated the correlations for all pairs of FAs and AAs over all RILs independently. We found strong correlations between different clusters of metabolites (Fig. 4). For AAs, we observed a strong positive correlation cluster between several hydrophobic AAs, including methionine and phenylalanine, the hydrophilic AA tyrosine, and branched-chain AAs (BCAAs) valine, leucine, and isoleucine (Fig. 4A). Another positively correlated cluster was found in ornithine, citrulline, glycine, serine, lysine, glutamic acid, and aspartic acid (Fig. 4A). This cluster negatively correlated with the former positive cluster. Overall, we observed correlations between many metabolites, suggesting shared genetic variants that regulate the levels of these metabolites.

In the FA correlation profile, we observed a strong positive correlation in several polyunsaturated FAs (PUFAs), including C18:3, C20:3, C20:4, and C20:5 forming a small cluster (Fig. 4B). Strong correlations were also found in a large cluster of long-chain and very-long-chain FAs. Another large cluster was formed by C30:0 and several unsaturated FAs (e.g., C20:1, C22:1, C24:5, and C24:6) (Fig. 4B). Notably, these last two clusters showed a strong negative correlation with the first small cluster. Taken together, the strong correlations within metabolite classes imply that linked metabolite QTL (mQTL) could be detected as many metabolites show similar patterns of variation over the RILs.

### Multiple QTL link metabolite levels to causal loci

**Figure 5.**
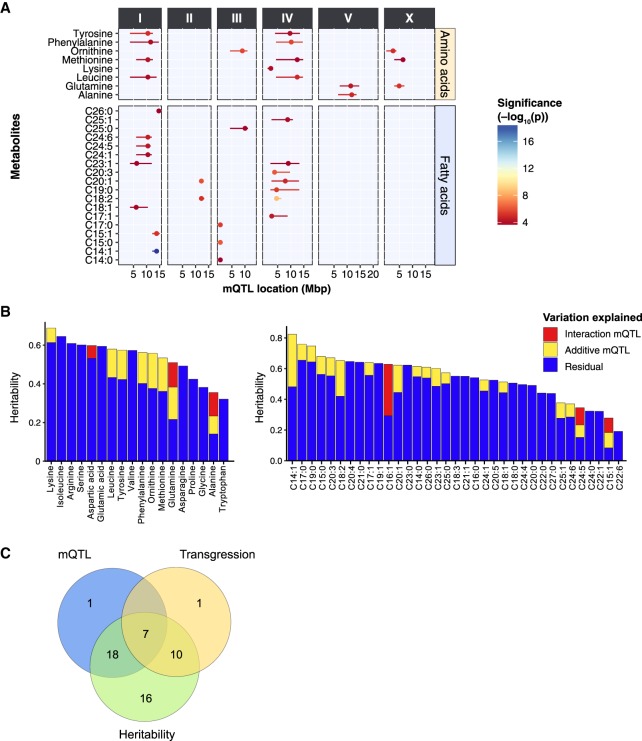
QTL mapping of metabolite levels in the 199 RILs. (*A*) To identify genetic factors responsible for the observed metabolite trait variations, we performed QTL mapping with the metabolite profiles of 199 RILs. In total, 36 significant mQTL were detected for AA and FA traits at an FDR = 0.05 [−log(*p*) > 3.7], ranging from 4.2 to 16.5 (Supplemental Table S7). The *x*-axis displays the position of the QTL in mega base pairs (Mbp) for each chromosome, and the *y*-axis displays the trait for which a significant QTL was found. (*B*) The heritability of each metabolite and the amount of variation explained by identified mQTL. Two classes of mQTL are distinguished: additive loci and locus–locus interactions. (*C*) Overlapping metabolite traits that are identified in QTL mapping, heritability analysis, and transgressive segregation analysis. Except for one metabolite trait (C15:1), all metabolite traits that were mapped to one or more significant QTL were highly heritable. Seven transgressive metabolite traits appeared to be associated with one or more significant QTL. These seven traits also have significantly higher heritability.

To identify loci that explain variation in metabolite levels, we performed QTL mapping on 56 metabolites measured in the 199 RILs (Fig. 5A). We expected to detect 80% of the genomic loci explaining a minimum of 10% per locus of the trait variation in this population when using a single-marker model with the −log_10_(*p*) > 3.7 as the significance threshold (Supplemental Table S5). We detected 36 significant mQTL for 26 metabolites (Fig. 5A; Supplemental Table S6), which shows that specific loci affecting metabolite trait variation can be identified. We observed 15 significant mQTL for eight different AAs. We found that four AAs (tyrosine, phenylalanine, methionine, and leucine) shared two broad mQTL peaks on Chromosome I (∼10.5 Mbp) and Chromosome IV (∼11.0 Mbp). These four AAs also displayed strong positive correlations between the trait levels in the RILs (Fig. 4A). For the AA methionine, an additional mQTL was found on Chromosome X. Individually, each of these three QTL explained ∼7% of the total variation, and together they explained 18.5% of the variation in methionine levels between the RILs in an additive model. Furthermore, only two AAs, lysine and alanine, were each mapped to a single locus, on Chromosomes IV and V, respectively. For the FA traits, we detected a total of 21 significant mQTL for 18 unique FAs. Among these FAs, we observed several shared mQTL. For instance, we detected a mQTL in the first 0.6 Mbp of Chromosome II for C14:0, C15:0, and C17:0. Also, the mQTL for three very-long-chain FA species—C24:1, C24:5, and C24:6—were all mapping to a locus on Chromosome I, with a peak at 10.5 Mbp. Across the QTL analysis, the most significant mQTL we detected [−log_10_(*p*) = 18.8] was for C14:1, which mapped to a single locus on Chromosome I within a region of 12.2–14.7 Mbp. This metabolite trait also had the highest heritability (*H*^2^ = 0.82), without significant transgression.

### Epistatic interactions between mQTL and genetic complexity

In order to investigate whether epistasis played a role in the metabolite levels, we took two approaches. First, we tested for interactions between the mQTL peaks of the nine metabolites with multiple peaks, but failed to identify strong interactions (*P* > 0.01) (Supplemental Table S7). Second, we conducted a genome-wide two-marker scan for interactions. We identified 17 locus–locus interactions for six metabolites: C15:1, C16:1, C24:5, alanine, aspartic acid, and glutamine (FDR < 0.1) (Supplemental Table S7). Combining the single-marker and two-marker scan mQTL models, we calculated the amount of heritable variation explained by these mQTL. For some metabolites, such as isoleucine, no variation could be explained despite a high heritability. In contrast, for C14:1 ∼50% of the heritability was explained by mQTL. In total, for 28 metabolites part of the *H*^2^ could be explained by the identified mQTL (Fig. 5B).

To gain an overview of the genetic complexity and its effect on the detection of QTL, we identified the overlap between metabolite traits with significant mQTL, transgressive segregation, and heritability (Fig. 5C): 7/18 metabolite traits (39%) that display significant transgression also mapped to significant mQTL, and 17/18 metabolite traits (94%) that have significant transgression also had relatively high heritability. Furthermore, 27/51 metabolite traits (53%) that showed a moderate to high heritability were mapped to one or more significant mQTL. Overall, a negative relation was observed between the number of transgressive strains and the successful mapping of mQTL (Supplemental Fig. S1). These findings suggest that many metabolite traits showed heritable variation and the genetic regulation of these metabolite traits was highly complex and likely involved multiple interactions between different genetic variants. This was supported by an analysis of the heritable variation in the parental strains versus the *H*^2^ in the RILs (Supplemental Fig. S2). Here we observed that most of the *H*^2^ was driven either by many additive loci with opposing effects or by genetic interactions such as epistasis.

### Independent confirmation of the C14:1 and C18:2 mQTL using introgression lines

**Figure 6.**
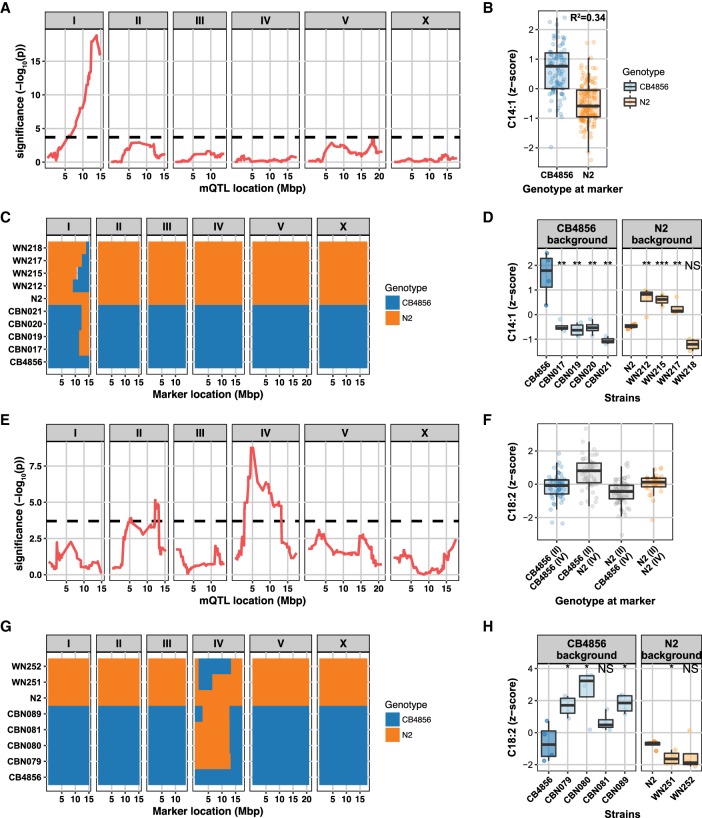
QTL peaks for C14:1 and C18:2 levels and narrowing down the QTL peak regions with ILs, respectively. (*A*) A strong QTL for C14:1 was detected on Chromosome I (the dashed line indicates FDR = 0.05 threshold). (*B*) The genetic variations attributed to a QTL were calculated by the correlation between the metabolite level and the genotype at the peak location. RILs that have a CB marker at this locus have a relatively higher abundance of C14:1 compared to those that have an N2 marker. Thirty-four percent of the variation in FA C14:1 levels can be explained by genetic variation on Chromosome I. (*C*) Genotypes of the ILs with either a CB4856 background or an N2 background. The genome of an IL is composed of a recipient genome contributed by one of the parental strains and a short homozygous segment of the genome contributed by the other parental strain. WN212, WN215, WN217, and WN218 strains are ILs with an N2 background, and CBN017, CBN019, CBN020, and CBN021 are ILs with a CB4856 background. Genomic segments from N2 are marked with orange; those from CB4856 are marked with blue. (*D*) Metabolite profiles of ILs and two parental strains. CB4856 has higher levels of C14:1. All CBN lines have lower levels than CB4856 (metabolite levels were normalized to percentage *z*-score), meaning that there is a QTL covered by all of these lines; same for all WN lines, only WN218 has a lower level than N2. Therefore, this strain does not contain the QTL. The WN ILs narrow the QTL for C14:1 down to a region of 12.42–13.84 Mbp. (*E*) Two QTL were detected for C18:2: One QTL peak was detected on Chromosome II and one on Chromosome IV (the dashed line indicates FDR = 0.05 threshold). (*F*) RILs that have an N2 marker at the locus on Chromosome IV have a relatively higher abundance of C18:2 compared to those that have a CB4856 marker. Twenty-seven percent of the variation in the FA C18:2 level can be explained by genetic variation on Chromosomes II and IV. (*G*) Genotypes of the ILs with either a CB4856 background or an N2 background. CBN079, CBN080, CBN081, and CBN089 are ILs with a CB4856 background, and WN251 and WN252 strains are ILs with an N2 background. Genomic segments from N2 are marked with orange; those from CB4856 are marked with blue. (*H*) FA profiles of ILs and two parental strains. Three of the CBN lines have higher levels than CB4856 (metabolite levels were normalized to percentage *z*-score), and one of the two WN strains confirmed the mQTL for C18:2. The mQTL could be confirmed to a region of 2.8–6.4 Mbp. Significance was calculated using Student's *t*-test and corrected for multiple testing. (*) *q* < 0.05; (**) *q* < 0.01; (***) *q* < 0.001; (NS) not significant.

Next, we decided to focus on two mQTL that displayed the most significant linkage and high heritability: the mQTL for C14:1 on Chromosome I and the one for C18:2 on Chromosome IV. For C14:1, 34% of variation could be explained by the genotype at the peak location on Chromosome I (Fig. 6A,B), and RILs with a CB4856 genotype at this QTL were associated with higher levels of C14:1 than RILs with an N2 genotype at this QTL (Fig. 6B). This shows that a major locus is affecting C14:1 levels between these strains.

We next used introgression lines (ILs) to independently validate the C14: 1 mQTL and narrow down the mQTL peak to a smaller region. These ILs contain a small genomic segment derived from one parental strain introgressed into the genetic background of the other parental strain (Fig. 6C). For C14:1, we used eight ILs (Sterken 2016). Four strains (WN212, WN215, WN217, and WN218) contain a CB4856-derived introgression in an N2 genetic background (Doroszuk et al. 2009), and four strains (CBN017, CBN019, CBN020, and CBN021) contain an N2-derived introgression in a CB4856 genetic background (Sterken 2016). The introgressions of both IL sets cover the C14:1 mQTL on Chromosome I (Fig. 6C). We measured the metabolite levels in the ILs and tested the hypotheses put forward from the RIL experiment (Fig. 6D; Supplemental Fig. S3): (1) An N2 locus decreases the C14:1 abundance, and (2) a CB4856 locus increases the abundance. We found that the four CB4856-background ILs had lower C14:1 levels, confirming the first hypothesis (FDR < 0.05). Three out of four N2-background ILs had increased C14:1 levels, which confirmed the second hypothesis (FDR < 0.05). WN218 did not show a significant increase in the C14:1 abundance (Fig. 6D). By analyzing the introgression locations, we hence narrowed down the mQTL region to 12.4–13.8 Mbp.

For the FA C18:2, two QTL were identified: one on Chromosome II (lower abundance in N2 genotype) and one on Chromosome IV (higher abundance in N2 genotype) (Fig. 6E,F). Together, these two loci explain 27% of the trait variation. For confirmation of the C18:2 mQTL, we used two N2 genetic background ILs (WN251 and WN252) and four CB4856 genetic background ILs (CBN079, CBN080, CBN081, and CBN089) (Fig. 6G). As for C14:1, we tested the prediction from the IL panel: an increase in abundance by the CB4856 locus and a decrease by the N2 locus (in presence of the minor mQTL) (Fig. 6H; Supplemental Fig. S4). Here, three of the four CB4856-background ILs and one of the two N2-background ILs confirmed the mQTL for C18:2 (FDR < 0.05). Based on the minimum region of the confirming ILs, the mQTL could be confirmed to the region spanning 2.8–6.4 Mbp. Overall, these results independently confirmed the detection of the two mQTL in the RILs, demonstrating that this approach is an important step to make forward genetics possible for identifying complex genetics underlying metabolic regulation.

### Identification of candidate genes in the Chromosome I mQTL

In order to identify candidate genes that might underlie the variation in metabolite levels, we performed a candidate gene approach on the Chromosome I mQTL, because this was the most significant QTL but also because three FAs mapped to the same locus. A computational approach was used to prioritize candidate genes by compiling a list of polymorphic genes that are (1) involved in lipid metabolism or (2) transcription factors (Thompson et al. 2015; Kanehisa et al. 2016; Lee et al. 2018). These were cross-referenced with the single-marker mQTL peaks. Candidates were prioritized based on polymorphisms that lead to an aberrant protein or potentially differential regulation (Supplemental Table S8). Next, we analyzed C14:1 profiles of N2 worms with RNAi against a selection of 19 genes from all the candidates. The C14:1 amounts in RNAi-treated worms were compared to worms treated with control RNAi (*Escherichia coli* HT115 strain) (Supplemental Fig. S5). Of all 19 candidates tested, we found that worms fed with RNAi targeting *lagr-1*, Y87G2A.2, *nhr-265*, *nhr-276*, and *nhr-81* contained a significantly higher amount of C14:1 compared to the control (Supplemental Fig. S5A,B). The mammalian homolog of *lagr-1* is CERS1, which encodes ceramide synthase 1 catalyzing the synthesis of C18 (dihydro)ceramide (Zhao et al. 2011). It was previously shown that worms with reduced expression of *lagr-1* have a reduced lipid content using Nile Red staining (Ashrafi et al. 2003). Y87G2A.2 encodes an enzyme that functions similarly to mammalian acyl-CoA thioesterase 8, which catalyzes the hydrolysis of acyl-CoA to free FAs in peroxisomes (Hunt and Alexson 2002). Although transcription factors *nhr-265*, *nhr-276*, and *nhr-81* have never been studied on their roles in lipid metabolism, our findings provide potential novel functions of these transcription factors that could be explored in future work. Because the long-chain FA C15:1 and the very-long-chain FA C26:0 also mapped to the same Chromosome I locus, we also measured the amount of C15:1 and C26:0 in the same RNAi-treated samples (Supplemental Fig. S5C,D). Worms fed with RNAi against *acox-1.1* or *scrm-2* showed significantly decreased C15:1 amounts, suggesting a causal role of these two genes in the regulation of lipid abundance (Supplemental Fig. S5C). The C26:0 concentration increased when *acox-1.1* or *acox-1.4* (isoforms of the same gene) was knocked down (Supplemental Fig. S5D). This is fully in line with the suspected biochemical role of these proteins, since these are homologs of human *ACOX1* encoding peroxisomal acyl-coenzyme A oxidase 1. This enzyme catalyzes the first reaction of the very-long-chain FA oxidation pathway in peroxisomes (Wanders and Waterham 2006).

## Discussion

Imbalanced metabolic homeostasis often leads to chronic metabolic diseases (Gao et al. 2014). How genetic and environmental factors contribute to disturbed metabolism differs significantly among individuals and this process remains elusive (Andreux et al. 2012). Recent technological advances in “-omics” studies have made it possible to measure large-scale interactions in many components of a cell, allowing the study of complex biological systems and identification of new biochemical mechanisms (Williams et al. 2016). An example of such work is a GWAS on human blood metabolites collected from two European population studies (Shin et al. 2014). Among many associations, there was a striking association between the *SCD* gene and the FA myristoleate C14:1 (Shin et al. 2014). Although mammalian model systems traditionally serve as a platform to identify association between genetic variants and metabolic consequence, it can be too complex to dissect the underlying mechanism of action in complex metabolic traits. Hence, we chose to use the genetically tractable and cost-effective organism *C. elegans* in this study. To study the quantitative trait metabolite abundance, we measured FAs and AAs in a population of 199 *C. elegans* RILs derived from the genetically diverse strains N2 (Bristol) and CB4856 (Hawaii) (Li et al. 2006; Thompson et al. 2015; Gao et al. 2017a). This systems approach enabled us to investigate linkage between genetics and metabolism, identifying genomic regions contributing to genetic variation in metabolite abundances.

In hybrids of inbred lines and crosses between populations of diverse animals, transgressive or extreme phenotypes (transgressive segregation) falling beyond the parental phenotypes are often seen (deVicente and Tanksley 1993; Rieseberg et al. 1999). For example, in *C. elegans* transgression has been reported for some quantitative morphological traits, such as body size, egg size, and fertility (Gutteling et al. 2007; Kammenga et al. 2007). For metabolite abundances, we found that 28.6% (18/63) of the metabolite traits displayed transgression. This suggests that the genetic variation underlying metabolite abundance is a complex trait. Transgressive segregation in metabolite abundances is lower compared to the levels found in yeast 41% (14/34), but more extreme compared to transgression in gene expression in *C. elegans* (affecting ∼6% of the genes) (Viñuela et al. 2012; Breunig et al. 2014). This makes sense in the light of metabolite levels as complex cellular phenotypes that can be affected on many regulatory levels. We then calculated the broad-sense heritability to estimate the genetic contribution to the high levels of variation we observed in the metabolite traits between the RILs. The majority of metabolite traits were highly heritable, suggesting there was a high chance of identifying an associated genomic locus. Previous studies in RILs from other organisms have also demonstrated that most metabolic traits are highly heritable (*H*^2^ > 0.5) (Andreux et al. 2012; Chen et al. 2014). Despite the fact that the heritability range differs across species, we showed ample evidence for moderate to high heritability in the metabolite traits of the 199 RILs with a range from 0.32 to 0.82, motivating us to identify detailed correlations between metabolites and the causal QTL for the metabolite traits.

Next, we analyzed the metabolite–metabolite correlations of the same metabolite classes and found strong correlations between multiple pairs of metabolite traits. Because involvement of different types of AAs in different metabolic pathways is rather complex, strong correlations between different pairs of AAs imply shared biological properties. All three BCAAs—valine, leucine, and isoleucine—showed strong positive correlation, likely because they share chemical similarities and are catabolized using similar pathways (Valerio et al. 2011; Mansfeld et al. 2015). In addition, it is worth noting that the first set of highly correlated AAs are all essential or derived from an essential AA (like tyrosine from phenylalanine). The remaining AAs are mostly synthesized from either sugar or TCA cycle intermediates. The source of AA synthesis as well as the different catabolic pathways may be relevant with respect to the observed anti-correlation. Two aromatic AAs tyrosine and phenylalanine were positively correlated together with all three BCAAs. Such correlation was previously observed in several type 2 diabetes studies in humans (Felig et al. 1969; Wang et al. 2011; Chen et al. 2016). For instance, in a nested case-control study for prediction of type 2 diabetes in a Framingham Offspring cohort, the abundance of these five AAs was significantly elevated under fasting state in high-risk individuals (Wang et al. 2011). Our finding that these five AAs strongly correlate suggests a conserved regulatory role. As metabolite traits are regulated in a complex fashion, QTL mapping highlights the complexity of genetic regulation of metabolite abundances (Andreux et al. 2012). To increase the power of our approach, we chose to measure metabolite profiles in a large number of strains instead of multiple biological replicates per strain, as this has the highest impact on QTL mapping power (Andreux et al. 2012). Out of 36 significant single-marker mQTL that mapped for 26 metabolite levels, nine metabolite traits were mapped to multiple mQTL, and some mQTL correlated with multiple metabolite traits. An additional two-marker mapping identified 17 pairs of interacting loci for six metabolite levels, showing that genetic interactions play a role in genetic variation underlying metabolites. Together, these findings indicate considerable genetic complexity in the regulation of metabolic quantitative traits (Flint and Mott 2001; Flint and Mackay 2009). To further investigate the genetic architecture of these quantitative phenotypes, we focused on the strongest mQTL detected for the FA C14:1 on Chromosome I. Using ILs that covered the genomic region of this mQTL, we were able to narrow it down to a 1.4-Mbp region. In genetic studies with segregating populations, such as hybrid populations of mice and flies, large numbers of QTL could be detected, although most of them have very small effects, and only a few loci have moderate to large effects on quantitative traits (Flint and Mackay 2009). In this study, the C14:1 mQTL served as an excellent example of a large-effect QTL as it showed a strong impact on the metabolite trait and explained large variations we have observed in the RILs, whereas the majority of the detected mQTL only had a small effect.

Independent confirmation of candidate genes by RNAi knockdown in Bristol N2 strain illustrated that *lagr-1*, Y87G2A.2, *nhr-265*, *nhr-276*, and *nhr-81* affected the levels of C14:1. Although *lagr-1* and Y87G2A.2 indeed play a role in lipid metabolism, the other genes, encoding transcription factors, have not been studied previously for their involvement in the regulation of lipid metabolism. This could support further research into detecting the causal polymorphic genes underlying the mQTL for C14:1. Since the scope of this study was to investigate the overall genetic architecture of metabolites in *C. elegans*, we did not pursue the search for causal genetic variants or to addressing the phenotypic effect of parental alleles of these candidate genes. Altogether, we believe that this RIL population of *C. elegans* provides us with a powerful platform to study the genetic basis of metabolism. The systems approach with QTL analysis makes it possible to address important questions related to genetic architectures of quantitative traits, such as genetic actions or gene-by-gene interactions and with the environment. The RILs can thereby play an important role to dissect the mechanisms underlying the complex processes of metabolism in a natural and unbiased manner and allow us to identify factors important for gene-by-environment interactions. In addition, this systems approach will also enable researchers to explore further into additional interventions, such as dietary alterations and environmental stresses–associated metabolic changes.

## Methods

### *C. elegans* strains and bacterial feeding strains

In total, 199 RILs were used (Li et al. 2006). Approximately twenty-five percent of these RILs have been genotyped by sequencing (Thompson et al. 2015). A list of the strain names and their genotypes can be found in the Supplemental Table (Supplemental Table S1). For narrowing down the range of mQTL on Chromosomes I and IV, 14 ILs were used. Six of these ILs had an N2 genetic background: WN212, WN215, WN217, WN218, WN251, and WN252 (Doroszuk et al. 2009). Eight of these ILs had a CB4856 genetic background: CBN017, CBN019, CBN020, CBN021, CBN079, CBN080, CBN081, and CBN089 (Sterken 2016). The sequenced genotypes of WN212, WN217, WN251, and WN252 have been published in Thompson et al. (2015); the sequenced genotypes of the strains WN218, and the CB4856 genetic-background strains are included in this article (Supplemental Table S9).

*E. coli* OP50 was obtained from the Caenorhabditis Genetics Center (CGC), RNAi bacterial clones are *E. coli* HT115 strains, including those to knockdown *acox-1.1*, *acox-1.3*, *acox-1.4*, *gpdh-1*, T27F6.6, *ech-7*, *lagr-1*, Y87G2A.2, *scrm-2*, *nhr-82*, *nhr-265*, *nhr-81*, *nhr-165*, *nhr-217*, *nhr-77*, *nhr-174*, *nhr-169*, *nhr-276*, and *ces-2*, which were obtained from the Ahringer library.

### Strain culturing and experiments

Nematodes were cultured and maintained at 20°C on nematode growth media (NGM) agar plates. Culture conditions in all experiments were the same unless indicated otherwise. For metabolite profiling of 199 RIL strains, N2, and CB4856, age-synchronized worms were obtained by alkaline hypochlorite treatment of gravid adults grown on *E. coli* OP50 lawn, 2000 eggs of each strain were then seeded onto NGM plates and cultured for 2.5 d, allowing development to young adults. For heritability analysis of FAs and AAs, we collected worms in triplicates from 51 RIL strains, in which the genome composition contains high recombination (together with N2 and CB4856). To narrow down the QTL peak for C14:1 on Chromosome I and the one for C18:2 on Chromosome IV, we prepared worm samples in four replicates for the ILs.

To investigate candidate genes, we measured FA profiles of N2 worms with RNAi of candidate genes. RNAi experiments were performed from hatch in all cases, on NGMi plates (containing 2 mM IPTG and 25 mg/mL carbenicillin). Two thousand Bristol N2 eggs were seeded onto NGMi plates with a bacterial lawn of either *E. coli* HT115 (RNAi control strain, containing an empty vector) or RNAi clones mentioned in the previous section. After 2.5 d, young adult worms were collected and freeze-dried until FA extraction.

### Whole-genome sequence library prep and analysis for CB4856-background ILs

DNA was isolated from 100 to 300 µL of packed worms using the Qiagen blood and tissue kit (catalog no. 69506). Following the ATL lysis step, 4 µL of 100 mg/mL RNAse was added to each sample and allowed to incubate for 2 min at room temperature. DNA concentration was determined using the Qubit dsDNA BR Assay Kit (Thermo Fisher Scientific; catalog no. Q32850). For each strain, a total of 0.75 ng of DNA was combined with 2.5 µL transposome (Illumina; kit no. FC-121-1011) diluted 35× in 1× Tris buffer (10× Tris buffer: 100 mM Tris-HCl at pH 8.0, 50 mM MgCl_2_) in a 10 µL final volume on ice. This reaction was incubated at 55°C for 10 min. The amplification reaction for each strain contained (final concentrations): 1× Ex Taq Buffer, 0.2 mM dNTPs, 1 U Ex Taq (Takara; catalog no. RR001A), 0.2 µM primer 1, 0.2 µM primer 2, and 5 µL of tagmentation material from the previous step in a 25 µL total volume. Each strain had a unique pair of indexed primers. We first made a master mix containing buffer, water, dNTPs, and Ex Taq and then aliquoted the appropriate volume of this mix into each well. We added the specific primer sets to each well and finally the tagmentation reaction. The amplification reaction was incubated in a thermocycler with the following conditions: 72°C for 3 min (1 cycle); 95°C for 30 sec (1 cycle); 95°C 10 sec, 62°C 30 sec, and 72°C 3 min (20 cycles); and 10°C hold. We combined 8 µL from each amplification reaction to generate a pool of libraries. A portion of the libraries was electrophoresed on a 2% agarose gel. DNA was excised and gel purified using Qiagen's gel purification kit (catalog no. 28706). The libraries were sequenced on the Illumina HiSeq 2500 platform using a paired-end 100-bp reaction lane. Alignment, variant calling, and filtering were performed as previously (Cook et al. 2016). CB4856-background IL genotypes were called using the VCF file and a hidden Markov model as described previously (Cook et al. 2016; Cook and Andersen 2017).

### Metabolomics—fatty acid extraction and MS analysis

Sample preparation for FA extraction was followed as mentioned in our previous study (Gao et al. 2017a). A synchronized population of 2000 young adults was washed off the plates in M9 buffer, and the worm pellet was washed with dH_2_O for three times and then collected in a 2-mL Eppendorf tube and freeze-dried overnight. Dried worm pellets were stored at room temperature until use. Dry worm pellets were resuspended in 250 µL ice-cold 0.9% NaCl solution and homogenized with a 5-mm steel bead using a TissueLyser II (Qiagen) for two times of 2.5 min at frequency of 30 times/sec, followed by a tip sonication (energy level: 40 joule; output: 8 watts) for two times on ice water. Protein quantification was performed with BCA assay.

Worm lysate (up to 150 µg protein) was transferred in a 4-mL FA-free glass vial, and 1 mL of freshly prepared 100% acetonitrile (ACN)/37% hydrochloric acid (HCl) (4:1, v/v) was added to the lysate, together with deuterium-labeled internal standards. FA samples were hydrolyzed by incubating at 90°C for 2 h. After the vials cooled down to room temperature, 2 mL of hexane was added to the samples and mixed by vortexing for 5 sec followed by a centrifugation step at 1000*g* for 1 min. The upper layer was transferred to an FA-free glass tube and evaporated at 30°C under a stream of nitrogen. FA residues were dissolved in 150 µL chloroform-methanol-water (50:45:5, v/v/v) solution containing 0.0025% aqueous ammonia and then transferred to a Gilson vial for ESI-MS analysis.

We added deuterium-labeled internal standards to each sample, including 17,17,18,18,18-D5-C18:0 (5.04 nmol, CDN isotope, Canada), 3,3,5,5-D4-C24:0 (2.52 nmol, Organic Synthesis Laboratory of VU Medical Center) and 3,3,5,5-D4-C26:0 (0.25 nmol, Organic Synthesis Laboratory of VU Medical Center); we also ran additional five samples with increasing amount of a FA mixture (C18:0, C24:0, and C26:0) to make a five-point calibration curve. In these additional five samples, above-mentioned internal standards were also added (Gao et al. 2017a).

### Metabolomics—amino acid extraction and UPLC-MS/MS analysis

We used the same worm homogenate as mentioned and prepared for FA analysis. As described previously, AAs were extracted by transferring worm lysate (contains 50 µg of protein) to a 2-mL Eppendorf tube, and 1 mL of 80% ACN plus 20 µL of internal standard mixture was added to the lysate and homogenized by vortexing (Gao et al. 2017a). Samples were centrifuged, and the supernatant was transferred to a 4-mL glass vial and evaporated under a stream of nitrogen at 40°C. After evaporation, AA residue was dissolved in 220 µL of 0.01% (v/v in MQ water) heptafluorobutyric acid. Then the suspension was transferred to a Gilson vial for UPLC-MS/MS analysis.

As standards, an internal standard mixture (Cambridge Isotope Laboratories) containing 68 nmol DL-alanine-2,3,3,3-D4, 44 nmol DL-glutamic acid-2,4,4-D3, 40 nmol L-leucine-5,5,5-D3, 28 nmol L-phenylalanine-ring-D5, 34 nmol L-valine-D8, 34 nmol L-methionine-(methyl-D3), 26 nmol L-tyrosine-ring-D4, 22 nmol L-tryptophan-(indole-D5), 46 nmol DL-serine-2,3,3-D3, 48 nmol proline-D7, 24 nmol L-arginine-2,3,3,4,4,5,5-D7, 28 nmol L-glutamine-2,3,3,4,4-D5, 32 nmol L-lysine-4,4,5,5-D4, 26 nmol L-citrulline-ureido-^13^C, 28 nmol L-ornithine-3,3,4,4,5,5-D6, 42 nmol L-isoleucine-D10, and 46 nmol DL-aspartic acid-2,3,3-D3 was added to each sample. For the calculation of AA concentrations, an additional sample was prepared by adding 50 µL standard mixture containing all AA with a concentration of 250 µmol/L to the internal standard (20 µL, same composition as mentioned above) and analyzed together with the RIL samples.

### Batch correction and data normalization

The 199 RILs were grown in five time-separated batches alongside with the parental strains N2 and CB4856. Metabolites were measured in five batches of FA and AA measurements. A subset of 51 RILs and the two parental strains were grown in three additional time-separated biological replicates and were measured in an additional batch of FA and AA measurements. In total, these experiments yielded 400 samples after quality control (enough input material). More specifically, FA and AA measurements for N2 and CB4856 were repeated seven times; seven RILs were repeated five times, 44 RILs four times, 27 RILs two times, and 121 RILs one time. There are two reasons for these numbers. First, the experiment was conducted on the 199 RILs (along with parental strains) and then replicated on a subset of 51 RILs (three additional biological replicates, along with parental strains). Second, for some samples in the experiment on the 199 RILs, we were not sure whether there was enough material. Therefore, these were repeated in another batch. However, after measuring FA and AA levels, the quality was found to be sufficient (and corresponding to the other sample); therefore, we found it most prudent to include both samples. For other samples, however, the initial sample did not provide enough material and had to be discarded. Details on FA and AA measurement batches and biological replicates are included in the raw data table (Supplemental Table S2).

Because of reliability detection limits of the MS platform, FA measurements with a concentration below 0.03 nmol/mg of protein were removed from the analysis, as were AA concentrations below 0.4 nmol/mg of protein (Gao et al. 2017a). In case of heritability estimates and mQTL mapping, only reliably detected metabolites were considered; those detected in more than 100 samples. Since the measurements of the FA concentrations and the AA concentrations were conducted in the same sample, the amount we measured was expressed as a ratio of the total composition. This was calculated independently for FAs and AAs using

$$R_{x,i}=\frac{M_{x,i}}{\sum_{i}^{M}},$$

where *R* is the fraction of metabolite *i* (one of 40 FAs or one of 19 AAs) of all the metabolites (either FAs or AAs) measured in sample *x*. *M* is the concentration of metabolite *i* of sample *x*.

The fractions were batch corrected by

$$R_{x,i,\mathrm{cor}}=R_{x,i}-({\bar{R}}_{x,\mathrm{batch}}-{\bar{R}}_{x,\mathrm{total}}),$$

where *R* is the is the fraction of metabolite *i* in sample *x*, of which the difference between the batch average and the total average is subtracted. For evaluation of the fold-differences in metabolite abundances, a batch correction on the untransformed metabolite abundances was conducted using the same formula with the absolute metabolite concentrations as input.

Thereafter, the metabolite levels were expressed as a *z*-score by

$$Z_{x,i}=\frac{R_{x,i,\mathrm{cor}}-\mu_{R,i}}{\sigma_{R,i}},$$

where *Z* is the *z*-score of metabolite *i* of sample *x*, and *µ* is the mean for that metabolite and *σ* is the standard deviation. This transformation was used in the further analysis. Outliers were removed if the trait value exceeded *µ* ± 2*σ as measured per strain.

### Statistical analysis

#### Software used

The data was analyzed in “R” (version 3.3.3, x64) using custom written scripts (R Core Team 2017). The code required for analysis is made available via GitLab (https://git.wur.nl/mark_sterken/Metabolomics) and as Supplemental Code. In the analysis, the tidyverse package was used for organizing the data (www.tidyverse.org); most plots were made using ggplot2 (Wickham 2009).

#### Correlation analysis

The traits were analyzed for correlation by calculating the Pearson correlation between metabolites (FAs and AAs independently) on the metabolite levels.

#### Transgressive segregation

Transgressive segregation was calculated as in Brem and Kruglyak (2005), which is a method also applied in a metabolomics study in yeast (Breunig et al. 2014). In short, to determine for which traits the RIL panel displayed transgressive segregation, we first calculated the mean trait value over all observations for the parental lines (separately for N2 and CB4856). Second, the standard deviation (σ) was calculated for the N2 and CB4856 observations separately; the pooled σ was used for transgression calculations. Transgressive segregation was calculated as the number of RILs of which the mean trait value exceeded *μ* ± 2*σ, where the parent with the lowest *μ* determines the low threshold and the parent with the highest *μ* determines the high threshold.

The significance of transgression was determined by permutation, where the trait values were randomized over the strain designations. Subsequently, the same test as described above was executed. The permutation was repeated 1000 times for each trait, where after the obtained values were used as the by-chance distribution; the FDR = 0.05 threshold was taken as the 50th highest value.

#### Heritability estimation

The heritability of the metabolite levels was calculated over a subset of RIL strains for which repeated measurements were conducted (51 RILs, *n* ≥ 3) and for metabolites that were consistently detected (more than 100 observations). Using an ANOVA explaining the metabolite variation over the strains, the broad-sense heritability was calculated as

$$H_{\mathrm{RIL}}^{2}=\frac{V_{\mathrm{strain}}}{V_{\mathrm{strain}}+V_{\mathrm{res}}},$$

where *H*^2^ is the broad-sense heritability, *V*_strain_ is the variation explained by strain, and *V*_res_ is the residual variation. The significance of the heritability was calculated by permutation, where the trait values were randomly assigned to strains. Over these permutated values, the variance captured by strain and the residual variance were calculated. This procedure was repeated 1000 times for each trait. The obtained values were used as the by-chance distribution, and an FDR = 0.05 was taken as the 50th highest value.

In the parental strains (*n* = 11 for both N2 and CB4856) the heritability was calculated by ANOVA, using

$$h_{P}^{2}=\frac{0.5\times V_{\mathrm{parent}}}{0.5\times V_{\mathrm{parent}}+V_{\mathrm{res}}},$$

where *h*^2^ is the heritability, *V*_parent_ is the variation explained by the parental genotypes, and *V*_res_ is the residual variation. The factor 0.5 corrects for the overestimation of the additive variation in inbred strains (Hegmann and Possidente 1981). The same permutation approach as for the broad-sense heritability was applied, taking the FDR = 0.05 threshold as significant.

#### Quantitative trait locus (QTL) mapping

QTL were mapped using custom scripts in R (Supplemental Code). The metabolite levels were fitted in a single-marker model,

$$Z_{i,j}=x_{j}+e_{j},$$

where *Z* is the *z*-score averaged over all strain replicates for metabolite *i* (one of 39 FAs or one of 19 AAs that were reliably measured in >100 strains) of RIL *j* (*n* = 199). This was explained over the genotype (either CB4856 or N2) on marker location *x* (*x* = 1, 2, …, 729) of RIL *j*.

For nine metabolites, multiple mQTL were identified with the single-marker model; to verify that these mQTL were not caused by linkage and were indeed independent, the mQTL peaks were fitted to an additive multiple marker model,

$$Z_{i,j}=x_{1,j}+x_{2,j}+\cdots +x_{n,j}+e_{j},$$

where *Z* is the *z*-score averaged over all strain replicates for metabolite *i* (one of three FAs with multiple mQTL: C18:2, C20:1, or C23:1; or one of six AAs with multiple mQTL: Gln, Leu, Met, Orn, Phe, or Tyr) of RIL *j* (*n* = 199). This was explained over the genotype (either CB4856 or N2) on marker location *x*_1_, *x*_2_, …, *x*_*n*_ (*x* = 1, 2, …, 729) of RIL *j*, which were identified in the single-marker mapping (maximum number of markers was three).

A full two-locus interaction model was fitted for all metabolites,

$$Z_{i,j}=x_{1,j}+x_{2,j}+x_{1,j}\times x_{2,j}+e_{j},$$

where *Z* is the *z*-score averaged over all strain replicates for metabolite *i* (one of 39 FAs or one of 19 AAs that were reliably measured in more than 100 strains) of RIL *j* (*n* = 199). This was explained over the genotype (either CB4856 or N2) on marker location *x*_1_, *x*_2_, and the interaction effect between *x*_1_ and *x*_2_ (*x* = 1, 2, …, 729) of RIL *j*. For the traits where a significant interaction was found (C15:1, C16:1, C24:5, Gln, Ala, and Asp), a full ANOVA model containing all the additive and interaction terms was used to determine the amount of variation explained by the additive and interaction terms. The effect sizes were calculated by a full linear model.

#### QTL threshold determination and power analysis

In order to account for multiple testing in the single-marker mapping, the genome-wide significance threshold was determined via permutation. Here the *z*-scores per metabolite were randomly distributed over the genotypes. This permutated data set was thereafter used in the same single-marker model for QTL mapping. This procedure was repeated for 100 randomized data sets. From these randomized mappings, an FDR was determined based on multiple testing under dependency (Benjamini and Yekutieli 2001),

$$\frac{\text{FDS}}{\text{RDS}}\leq \frac{m_{0}}{m}\times q\times \log(m),$$

where false discovery (FDS) is the outcome of the permutations and real discovery (RDS) is the outcome of the expression QTL (eQTL) mapping at a specific significance level. The value of *m*_0_, the number of true null hypotheses tested, was 56-RDS, and for the value of *m*, the number of hypotheses tested, the number of metabolites (56) was taken. The *q*-value was set at 0.05. In this way, a −log_10_(*p*) > 3.7 was found. The eQTL confidence interval was determined by a 1.5 drop in the −log_10_(*p*) value as measured from the eQTL peak.

The statistical power in the single-marker mapping at the FDR threshold was determined by simulation. Using the genetic map of the 199 strains used in this study, QTL were simulated for each marker location. Per location, 10 QTL were simulated, explaining 5%–80% of the variation (in increments of 5%). In order to simulate technical noise, we introduced random variation based on a standard normal distribution (*σ* = 1, *μ* = 0). The simulated peak size was set correspondingly (e.g., 50% explained variation corresponds to a peak size of two in the simulated noise). Based on the set permutation threshold [−log_10_(*p*) > 3.7], the number of correctly detected QTL, the number of false positives, and the number of undetected QTL were counted. From the simulation, we also inferred the precision of the mapping by evaluating the effect size estimation and the QTL location [based on a −log_10_(*p*) drop of 1.5]. The detailed results of the analysis can be found in Supplemental Table S5.

The significance threshold in the interaction model was determined on the marker–marker interaction with markers that were more than 50 markers apart (or located on different chromosomes). The p.adjust function in R was used to calculate the FDR.

#### Introgression line analysis

The ILs were grown in four time-separated biological replicates, which were measured in one batch of FA and AA measurements. The transformed metabolomics data on C14:1 and C18:2, obtained in eight ILs covering the C14:1 mQTL and six ILs covering the C18:2 mQTL, were compared to the genetic background parent. This means that the ILs with the CB4856 genetic background were compared to CB4856, and the ILs with the N2 genetic background were compared to N2. For both loci separately, we tested the differences in C14:1 and C18:2 abundance using a Student's *t*-test, explicitly testing the hypothesis formed from the RIL data. Therefore, for C14:1 the CB4856-background ILs were tested for lower trait levels compared to the genetic background strain, and the N2-background ILs were tested for higher trait levels compared to the genetic background strain. For C18:2 the CB4856-background ILs were tested for higher trait levels compared to the genetic background strain, and the N2-background ILs were tested for lower trait levels compared to the genetic background strain. The significances were adjusted for multiple testing using the Benjamini and Hochberg correction, as implemented in the p.adjust function in “R” (Benjamini and Hochberg 1995).

#### Candidate gene identification

The polymorphisms between N2 and CB4856 were obtained from the Supplemental Table of the CB4856 reference genome paper (Thompson et al. 2015). These were summarized per gene in three classes: (1) polymorphisms with a high impact on the coding sequence, (2) polymorphisms in the regulatory regions, and (3) polymorphisms with a low impact on the coding sequence. The first group consisted of nonsynonymous substitutions, stops gained or lost, exon deletions, in-frame insertions/deletions, frameshifts, and fully deleted genes. The second group consisted of polymorphisms in the 3′ and 5′ untranslated region, and presence in one of the highly divergent regions. The third group consisted of synonymous substitutions.

The metabolic pathways of FA synthesis were obtained from KEGG using the Bioconductor KEGGREST package (Kanehisa et al. 2017; http://bioconductor.org/packages/KEGGREST/). Specifically, the lipid metabolism pathways in *C. elegans* were obtained (Supplemental Table S10). This list was expanded by adding likely lipid metabolism genes identified in a comparative study on lipid metabolism genes in *C. elegans* (Zhang et al. 2013). As also regulatory genes could be potential candidates, the genes of *C. elegans* with an ontology term containing the word “transcription factor” were downloaded from WormBase (WS258) (Lee et al. 2018).

## Data access

The sequencing data for genotyping of the introgression lines have been submitted to the NCBI Sequence Read Archive (SRA; https://www.ncbi.nlm.nih.gov/sra) under accession number SRP154243. The MS data are available at the NIH Common Fund's Metabolomics Data Repository and Coordinating Center (supported by NIH grant, U01-DK097430) website, the Metabolomics Workbench, http://www.metabolomicsworkbench.org, where they have been assigned Project ID PR000676. The data can be accessed directly via their Project DOI: 10.21228/M8968S (https://doi.org/10.21228/M8968S).

## Supplementary Material

Supplemental Material
