# Supplementary material for: Natural genetic variation in C. elegans identified genomic loci controlling metabolite levels
Source: Genome Res. 2018 Sep;28(9):1296–308. doi: 10.1101/gr.232322.117 (PMC6120624; doi:10.1101/gr.232322.117)
Supplement: Supplemental Material [file supp_28_9_1296__index.html]

Natural genetic variation in C. elegans identified genomic loci controlling metabolite levels — Supplemental Material 

# Natural genetic variation in *C. elegans* identified genomic loci controlling metabolite levels

## Supplemental Material

- Supplemental\_Fig\_S1.docx
- Supplemental\_Fig\_S2.docx
- Supplemental\_Fig\_S3.docx
- Supplemental\_Fig\_S4.docx
- Supplemental\_Fig\_S5.docx
- Supplemental\_Table\_S1.xlsx
- Supplemental\_Table\_S2.xlsx
- Supplemental\_Table\_S3.xlsx
- Supplemental\_Table\_S4.xlsx
- Supplemental\_Table\_S5.xlsx
- Supplemental\_Table\_S6.xlsx
- Supplemental\_Table\_S7.xlsx
- Supplemental\_Table\_S8.XLSX
- Supplemental\_Table\_S9.xlsx
- Supplemental\_Table\_S10.xlsx
- Supplemental\_Code.zip
