# Supplementary material for: Natural genetic variation in C. elegans identified genomic loci controlling metabolite levels
Source: Genome Res. 2018 Sep;28(9):1296–308. doi: 10.1101/gr.232322.117 (PMC6120624; doi:10.1101/gr.232322.117)
Supplement: Supplemental Material [file supp_gr.232322.117_Supplemental_Fig_S1.docx]

**
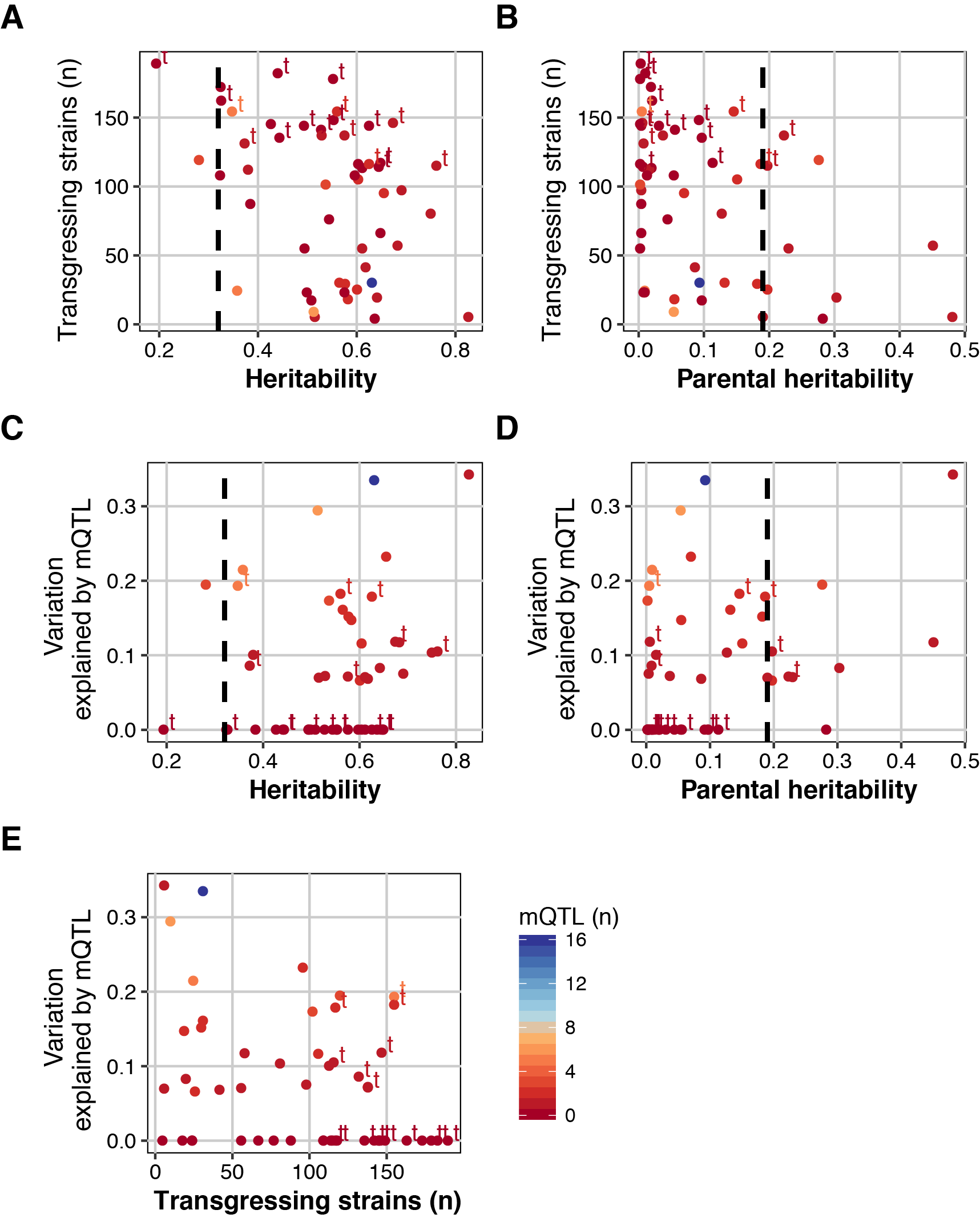
**

**Supplemental Figure S1. Transgression and heritability.**

(A) The number of strains that display transgression (n), versus the heritability (broad-sense, as calculated over the RILs). There was a significant negative association between the broad-sense heritability and the number of strains displaying transgression (ANOVA, p = 0.006). (B) The number of strains that display transgression (n), versus the parental heritability calculated over N2 and CB4856. There was a significant negative association between the parental heritability and the number of strains displaying transgression (ANOVA, p = 0.002). (C) The variation explained by mQTL (both additive and interactions) versus the broad-sense heritability. There was a negative association between the broad-sense heritability and the variation explained by mQTL (ANOVA, p = 0.05). (D) The variation explained by mQTL (both additive and interactions) versus the parental heritability. There was no association between the parental heritability and the variation explained by mQTL (ANOVA, p = 0.78). (E) The variation explained by mQTL (both additive and interactions) versus the number of strains that display transgression (n). There was no association between the parental heritability and the variation explained by mQTL (ANOVA, p = 0.23).
