# Supplementary material for: Natural genetic variation in C. elegans identified genomic loci controlling metabolite levels
Source: Genome Res. 2018 Sep;28(9):1296–308. doi: 10.1101/gr.232322.117 (PMC6120624; doi:10.1101/gr.232322.117)
Supplement: Supplemental Material [file supp_gr.232322.117_Supplemental_Fig_S2.docx]

**
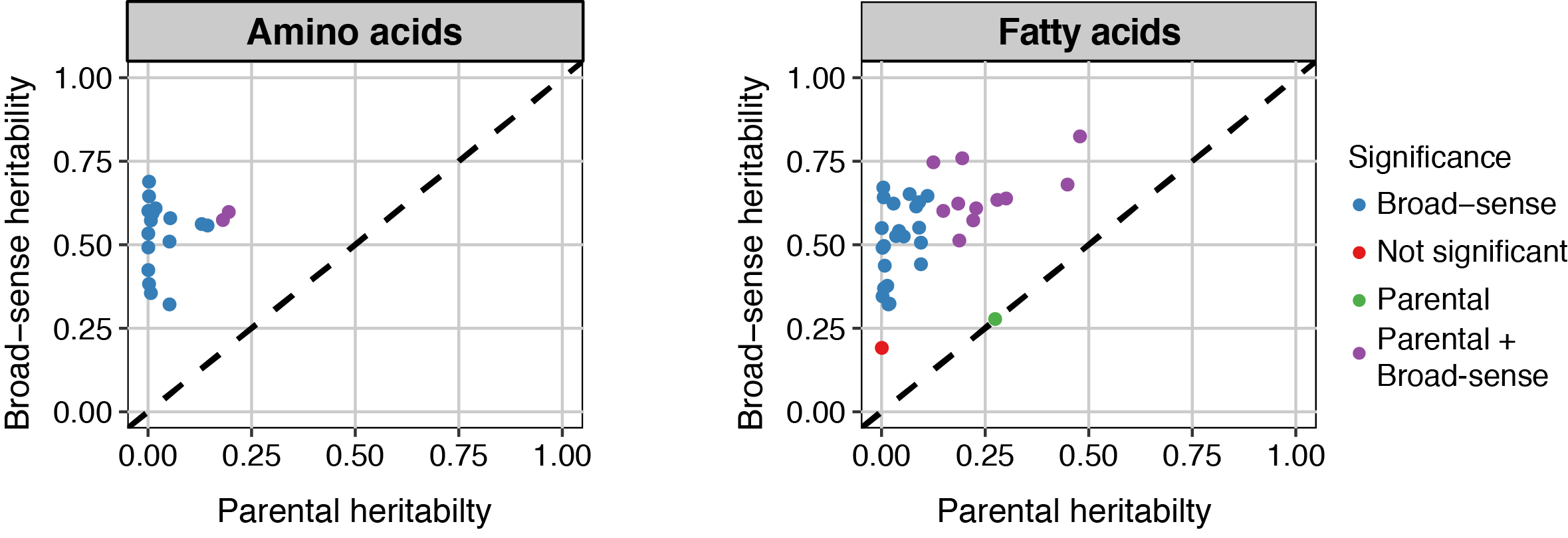
**

**Supplemental Figure S2. Heritability in the parental strains versus the broad sense heritability**

Plots of the heritability for both amino acids and fatty acids calculated in the parental strains versus the broad-sense heritability calculated in the RILs. The colors indicate the significance of the heritability estimated (as determined by permutation, false discovery rate < 0.05). If the broad-sense heritability exceeds the heritability in the parental strains, it indicates a complex trait architecture (multiple regulatory loci)
