# Supplementary material for: Natural genetic variation in C. elegans identified genomic loci controlling metabolite levels
Source: Genome Res. 2018 Sep;28(9):1296–308. doi: 10.1101/gr.232322.117 (PMC6120624; doi:10.1101/gr.232322.117)
Supplement: Supplemental Material [file supp_gr.232322.117_Supplemental_Fig_S3.docx]

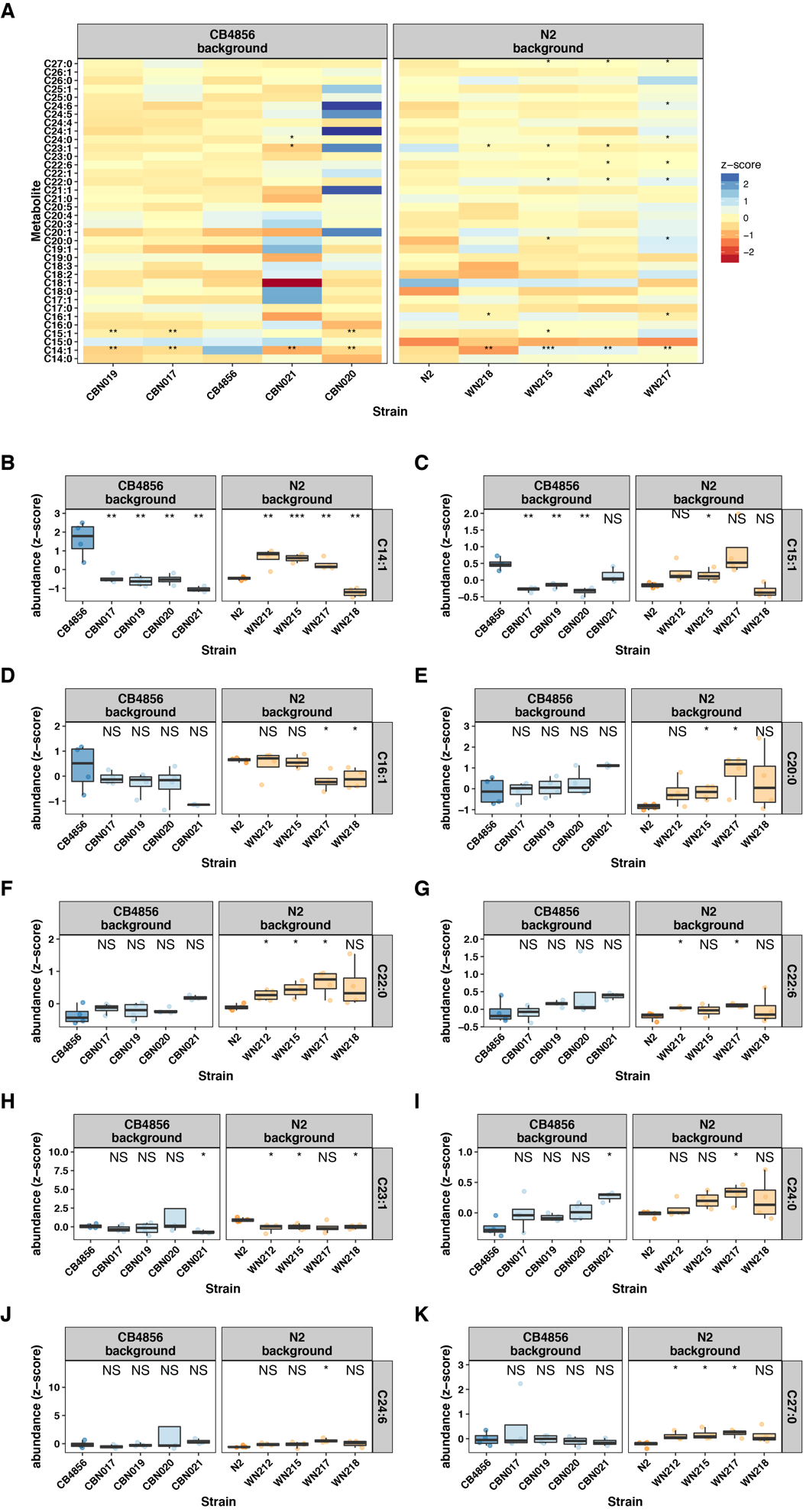


**Supplemental Figure S3. Chromosome I introgression lines**

(A) A heat map with the average metabolite levels for the fatty acids in the introgression lines covering chromosome I. The indicated significances are based on a two-sided Students t-test adjusted for multiple testing. NS, FDR > 0.05; *, FDR < 0.05; **, FDR < 0.01; ***, FDR < 0.001. (B-K) the measured values per IL for the metabolites with at least one significantly different strain (FDR < 0.05) in (A).
