# Supplementary material for: Natural genetic variation in C. elegans identified genomic loci controlling metabolite levels
Source: Genome Res. 2018 Sep;28(9):1296–308. doi: 10.1101/gr.232322.117 (PMC6120624; doi:10.1101/gr.232322.117)
Supplement: Supplemental Material [file supp_gr.232322.117_Supplemental_Fig_S5.docx]

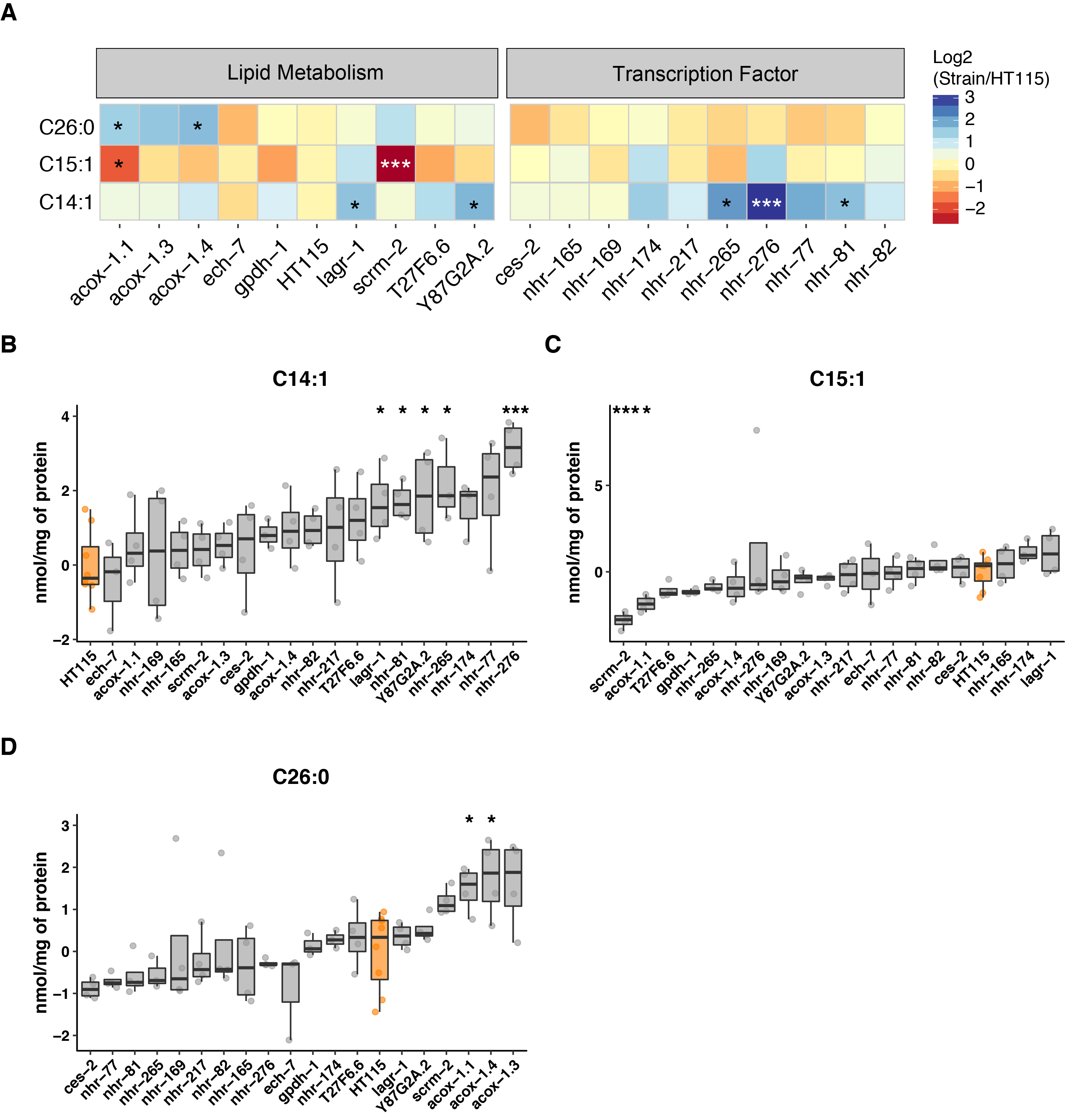


**Figure S5. Knock-down by RNAi for validation of candidate genes.**

(A) A heat map with the average log2 ratio versus HT115 for the fatty acids in the different RNAi treatments. (B-D) Level of C14:1, C15:1 and C26:0 in N2 worms with knock-down of candidate genes. Significance was calculated by comparing the measured fatty acid levels with empty vector fed worms (HT115) The significances indicated are based on a two-sided Students t-test adjusted for multiple testing. NS, FDR > 0.05; *, FDR < 0.05; **, FDR < 0.01; ***, FDR < 0.001. (FDR < 0.05).
